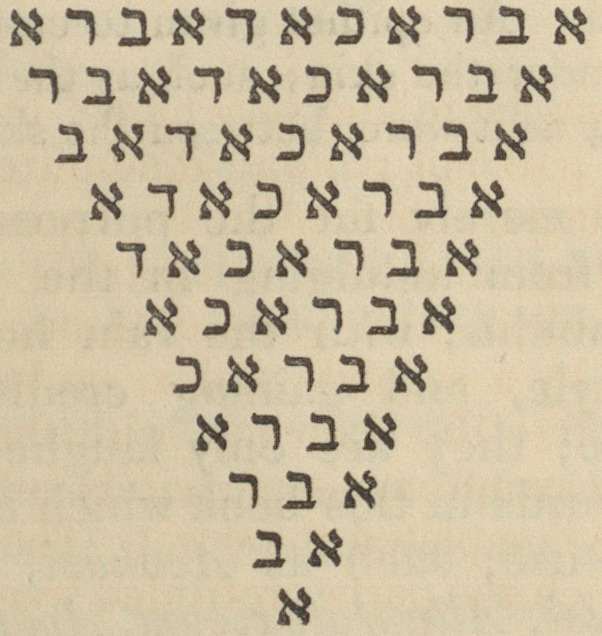# A New Dictionary of Medical Science and Literature

**Published:** 1833-10

**Authors:** 


*A New Dictionary of Medical Science and Literature: containing a concise Account of the various Subjects and Terms; with the Synonymes in different Languages, and Formulæ for various Officinal and Empirical Preparations, &c.* By ROBLEY DUNGLISON, M.D. Boston, 1833. Two Vols. 8vo. pp. 1239.

THIS compilation does credit to the industry of Dr. Dunglison, who is a professor in the university of Virginia; it contains an immense number of short and pithy explanations of scientific terms, which are rarely suffered to expand into an essay on the subject. In a few instances, however, as under the words Climate, Diet, and Feigned Diseases, the author has ventured to be more diffuse, and has, in general, made good use of the greater liberty he has allowed himself. A few extracts may give our readers some idea of the plan of the work.

"ABRACADABRA (*Med. Antiq.*), the name of a Syrian idol, according to Selden. This word, when pronounced and repeated in a certain form, and a certain number of times, was supposed to have the power of curing fevers, and preventing many diseases. It was figured on amulets, and worn suspended round the neck. The following description of it is given by Serenus Sammonicus, who had great faith in it.

"'Inscribas chartæ quod dicitur *Abracadabra*,
Sæpius et subter repetas, sed detrahe summæ,
Et magis atque magis desint elementa figuris
Singula, quæ semper rapies et cætera figes,
Donec in angustum redigatur litera conum.
His lino nexis collum redimire memento.'

אבראכאדאברא
אבראכאדאבר
אבראכאדאב
אבראכאדא
אבראכאד
אבראכא
אבראכ
אברא
אבר
אב
א

"Acclimatement, (F.) (*Hyg.*) The act of becoming acclimated, or accustomed to a climate. The constitution of a person who goes to live in another and very different climate, usually experiences changes, which are frequently of an unfavorable character, and the study of which is of considerable importance in medicine."

We should rather have said the *process* of becoming acclimated, than the *act*, as it does not take place at once.

"Anencephalus, (*Path.*) ανενκεφαλος, from α priv. and εγκεφαλος, brain. A monster devoid of brain. *Bonetus.* A weak, silly person. *Hippocr.*"

Our author, whose forte is certainly not in Greek, should have written ανεγκεφαλος. In another place, he derives that formidable word χολερα (cholera) from χολη, bile, and ρεω, I flow; but it is hardly necessary to say that χολερα, the nom. fem. of χολερος, is derived from χολη alone, and agrees with νουσος; the name of the malady at full length being ἡ χολερα νουσος, the bilious disease.

"The A'zygos Muscle. *A'zygos U'vulæ* is the small muscle which occupies the substance of the uvula. *Morgagni.* The name is however inappropriate, as there are two distinct fasciculi, placed along side each other, forming the *Paláto-staphylíni*, *Staphylíni*, or *Epistaphylini* muscles, *Staphylíni médii* of Winslow."

The eminent physician mentioned in the following article will smile when he reads it. Long may the latter blank remain unfilled!

"Bree, Robert, m.d. (*Biogr.*) A native of Warwickshire, and practised in London; he was born in ; died in . Works: Practical Inquiry on Disordered Respiration, &c. Lond. 1797, 8vo.; with an appendix, 1800, 8vo."

Dr. Dunglison is not quite *au niveau du siècle* in this little biography: there have been several editions of the work mentioned since 1800.

"CUTAM'BULUS (*Med.*), from *cútis*, skin, and *am'bulo*, I walk. Walking in the skin. An epithet given to certain parasitical animals which creep under the skin; such as the Guinea-worm, and to certain pains felt, as it were, between the skin and flesh."

We quote this merely for the purpose of warning our younger readers from indulging in the use of barbarous terms like Cutambulus, with the vain hope that they are adorning their style, and gaining credit for scholarship. Quite the reverse; they are only laughed at. There are many imaginary words in this book which happily have never been brought into use; such as *Aconusi*, *Adenochirapsologia*, *Æsthematonusi*, *Allantotoxicum*, *Amasesis*, *Amnioclepsis*, &c.

"DENG'UE (*Path.*) *Dingee*, *Danga*, *Dandy*, *Bouquet* and *Bucket Fever*. A disease which appeared, in the years 1827 and 1828, in the West Indies, and in the southern states of North America. It was extremely violent in its symptoms, but not often fatal. It usually commenced with great languor, chilliness, and pain in the tendons about the smaller joints. To these symptoms succeeded burning heat and redness of the skin, pains in the muscles of the limbs, or in the forehead, with vomiting or nausea. The fever continued for one, two, or three days, and usually terminated by copious perspiration. In different places it put on different appearances, but seems in all to have been a singular variety of rheumatic fever. The usual antiphlogistic treatment was adopted. and successfully.

"FÚGILE, (*Path.*) This term has several acceptations: it means, 1, the cerumen of the ear; 2, the nebulous suspension in, or the deposition from, the urine; 3, an abscess near the ear, (*Ruland* and *Johnson* ;) 4, abscess in general. *Forestus*."

"LY'TTA VITTA'TA, (*Mat. Med.*) *Cantharis vittáta*, *Potátoe Fly*. Four species of melöe that blister are found in the United States. The lytta vittata feeds principally upon the potatoe plant, and at the proper season of the year may be collected in immense number.

"The potatoe-fly resembles the cantharides in every property, and is fully equal to them.

"(F.) *Cantharide tachetée*."

"NOM'ADE, (*Anthropol.*) Nómas, νομας, from νομη, pasturage; an epithet given to people who have no fixed habitation, and who travel with their flocks from country to country, for pasturage.

Such are the Tartars. By analogy, the word Nomad'ic has been applied to spreading ulcers."

"Phlebecta'sia, (*Path.*), from φλεψ, a vein, and εκτασις, dilatation. Dilatation of a vein, or of a portion of a vein. *Alibert.*"

Alibert is much better employed in copying *dartres* than in coining Greek words, which seems to have become a sort of passion among the French.

"Quandros. Ancient name for a precious stone believed to exist in the brain of the vulture, and to which was attributed the property of augmenting the secretion of milk, and preserving from deadly accidents. No such stone exists."

A similar stone was supposed to exist in the head of the toad. Steevens, in his commentary on the well-known passage,

"Sweet are the uses of adversity," &c.

has quoted some directions from an old writer for ascertaining the genuineness of the toadstone: we are to show it to a toad, and, if it be "a ryght and true stone, the tode will leap towarde it, and make as though he would snatch it: he envieth so much that man should have that stone."

"Syphiloma'nia, (*Path.*), vulgarly *Noddle-pox.* A mania with which some persons are affected, so that they will subject themselves to antivenereal treatment, under the belief that they are affected with the syphilis of which they may have been previously cured."

These specimens will show the plan of Dr. Dunglison's Dictionary, which certainly, upon the whole, deserves considerable commendation.

## Figures and Tables

**Figure f1:**